# Liposomal drug delivery of *Aphanamixis polystachya* leaf extracts and its neurobehavioral activity in mice model

**DOI:** 10.1038/s41598-020-63894-9

**Published:** 2020-04-24

**Authors:** Mohammad H. Shariare, Mahbubur Rahman, Shamshad R. Lubna, Reeti S. Roy, Joynal Abedin, Akbar L. Marzan, Mohammad A. Altamimi, Syed Rizwan Ahamad, Ajaz Ahmad, Fars K. Alanazi, Mohsin Kazi

**Affiliations:** 1 Department of Pharmaceutical Sciences, North South University, Dhaka, Bangladesh; 2 Department of Pharmaceutics, College of Pharmacy, King Saud University, Riyadh, Kingdom of Saudi Arabia; 3 Central Laboratory, College of Pharmacy, King Saud University, Riyadh, Kingdom of Saudi Arabia; 4 Department of Pharmaceutical Chemistry, College of Pharmacy, King Saud University, Riyadh, Kingdom of Saudi Arabia; 5 Department of Clinical Pharmacy, College of Pharmacy, King Saud University, Riyadh, Kingdom of Saudi Arabia

**Keywords:** Alzheimer's disease, Parkinson's disease

## Abstract

Neurodegenerative diseases (Alzheimer’s, Parkinson’s etc.) causes brain cell damage leading to dementia. The major restriction remains in delivering drug to the central nervous system is blood brain barrier (BBB). The aim of this study was to develop a liposomal drug delivery system of *Aphanamixis polystachya* leaf extract for the treatment of neurodegenerative diseases such as Alzheimer’s and Parkinson’s disease. In this study GC-MS analysis is used to determine major constituents of *Aphanamixis polystachya* leaf extract. Liposomal batches of *Aphanamixis polystachya* leaf extract was prepared using design of experiment (DoE) and characterized using Malvern zetasizer, transmission electron microscopy (TEM), and FT-IR. Stability study of blank and leaf extract loaded liposome were performed in gastric media. *In-vivo* neurobehavioral and anti-inflammatory studies were performed on mice and rat model respectively. GC-MS data showed that major constituents of *Aphanamixis polystachya* leaf extract are 2-Pentanone, different acids (Octadec-9-enoic acid, 5-Hydroxypipeloic acid etc.), and Beta-Elemene etc. Malvern Zetasizer and TEM data showed that liposome batches of *Aphanamixis polystachya* leaf extract were in the range of 120 - 180 nm. Interactions between process parameters and material attributes found to have more impact on the average particle size and polydispersity of liposome batches compared to the impact of each parameter in isolation. Stability studies data suggest that blank and leaf extract loaded liposomes were stable at gastric conditions after 4 hours. *In-vivo* neurobehavioural study data indicated that significant improvement in the memory function, locomotor activity and ambulatory performance of dementia induced mice was observed for the liposomal batches compared to merely *A. polystachya* leaf extract.

## Introduction

One of the most researched areas in medical science is neuropharmacology. About 1.5 billion people worldwide are suffering from central nervous system diseases and nearly half of adults above 70 years old are expected to develop neurodegenerative diseases such as Alzheimer’s and Parkinson’s^1^. Dementia is a degenerative CNS condition that affects intellectual abilities and memory. Alzheimer’s, Huntington’s, and Parkinson’s, which are neurodegenerative diseases, can cause damage to different sets of brain cells leading to dementia^2–4^. Therefore, degenerative conditions, brain infections and stroke do alter the blood-brain barrier (BBB) causing foreign molecules to travers BBB and induce inflammation. However, altered BBB could be utilized as a mean for drug delivery into the brain. Currently, most therapies are symptomatic^5^, which make room for improvement in drug delivery system and potential discovery of new molecules. At present many CNS drugs such as tacrine, rivastigmine, donepezil, are cholinesterase inhibitor are used for the treatment of Alzheimer’s disease. Their main restriction, however, remains in the proper delivery to target region due to the BBB^6–8^.

To manage this dilemma, two different approaches are used for CNS drug delivery: invasive and non-invasive methods. However invasive methods are expensive, and there is an increased risk of infection when BBB is exposed^9^. Invasive methods can be used for well-defined tumors but not suitable for the disease like Alzheimer’s disease (AD)^7^. A non-invasive method like nanotechnology recently has received much attention for the treatment of different CNS diseases due to its targeted drug delivery^5,10,11^.

Phytoconstituents are used to treat cognitive disorders such as AD, Dementia and Parkinson’s disease through different approaches including acetylcholinesterase (AChE) inhibition, enhancement of cholinergic activity in CNS, anti-inflammatory, antioxidant and estrogen replacement therapy^12–15^. Previous research in ethnopharmacology lead to identifying some potential AChE inhibitors from plant sources including those for memory disorders (Dementia) and neurodegenerative diseases such as AD^12^^16–21^.

One such medicinal plant containing chemical components with likely therapeutic effects is *Aphanamixis polystachya* (Wall.) R. Parker belongs to the Meliaceae family. Phytochemicals of this plant are related to antioxidant, antimicrobial, anti-diarrheal, thrombolytic, diabetes and cytotoxic activities^22–25^. CNS depressant and analgesic activities were observed for the methanol extract of *A. polystachya* leaf^26^; however, to the best of our knowledge no drug delivery system has been utilized to improve the performance of the *A. polystachya plant* extracts and its activity against dementia.

The problem with herbal drugs is that they are poorly soluble in water leading to reduced bioavailability and escalated systemic clearance^27,28^. The other problem is the physical and chemical stability of the phytochemicals^27^^29–32^. Liposomes have been in use to improve the bioavailability of different phytoconstituents^33,34^. The strength of the liposome to interact with the body’s membrane is one of the key reasons as to why this delivery system was preferred over other nano drug delivery systems. Studies have deduced that the liposome doesn’t pass through the blood-brain barrier; it binds with the membrane instead, and hence allows the drug to cross through the other side once the liposome opens up^35,36^. Therefore, a systematic approach, i.e. Design of Experiment (DoE) has been used to shed some light on liposomal preparation process to identify various formulations and processing factors affecting the quality attributes of the liposome.

This work aimed to develop a liposomal drug delivery system of *A. polystachya* leaf extracts for the treatment of dementia and the probable mechanism of actions.

## Materials and Methods

### Materials

The leaves of the plant *Aphanamixis Polystachya* were collected from Dinajpur, Bangladesh. All solvents used in this study were purchased from Sigma Aldrich, India and was of analytical grade. Phospholipid was extracted from chicken egg yolk in-house. Cholesterol used was supplied by Sigma Aldrich, India. Olanzapine was obtained as a gift from Incepta Pharmaceuticals Ltd., Bangladesh. Carrageenan powder was purchased from Sigma Aldrich, Germany. Diclofenac sodium, 0.9% saline and disposable syringes were obtained from the Department of Pharmaceutical Sciences, North South University, Bangladesh.

### Drying and pulverization

The fresh leaves of the plant *Aphanamixis polystachya* were washed with water to remove adhering dirt. Then the leaves were cut into small pieces and separated. Finally, the leaves were dried for seven days. After complete drying, the leaves were pulverized into a coarse powder with the help of a grinding machine and were stored in an airtight container for further use. The ground powder was used to prepare extract by maceration method.

### Extraction (maceration method)

120 g of finely ground crude powder was used during each extraction process. The conical flask was rinsed with ethanol and 30 g of powder was taken in each 2 L clean dry conical flask. 1.5 L ethanol was added in each flask and then placed on a digital shaker for 7 days. Filter cloth was used to separate liquid portion. The liquid extract content was then evaporated using rotary evaporator at 40 °C and then left at room temperature to cool and solidify.

### Phospholipid extraction

Egg yolk was dissolved in a solvent mixture containing dichloromethane and methanol in a ratio of 1: 2. After 10 minute. the mixture was filtered and taken in a separatory funnel with an equal volume of 1% NaCl solution for separation. Hydroquinone, an anti-oxidant, was added to the collected bottom layer of the mixture and evaporation of the solvent was done by heating at 45 °C until a sticky yellow precipitate was observed. Then, precipitate was kept in an ice bath for 2–3 minute. and acetone was added. The portions not containing the phospholipids were dissolved in acetone and removed by filtration. The resulting phospholipid was stored at 4 °C.

### Design of experiment (DoE)

Liposomal batches of *Aphanamixis polystachya* were prepared using the design of experiment (DoE) with four factors. Each factor was studied at two levels; high and low. (Injection rate: Low: 0.25 mL/sec, High: 0.5 mL/sec, Stirring speed: Low: 750 rpm, High: 1500 rpm, Cholesterol:Phospholipid Low: 1:3 and High: 1:4.5, Drug in Solvent: Low: ethanol and High: water). The impact of the process parameters and material attributes on liposome-encapsulated drug delivery system (average size, and polydispersity index) was statistically evaluated using Minitab Version17.0 software. A full factorial design was employed to investigate the effects of various parameters on the characteristics of liposome encapsulated drug delivery system (Table 1).

**Table 1:** Design of Experiment of Liposomal batches of *Aphanamixis polystachya*.

Sample	Injection Rate (mL/sec)	Stirring Speed (rpm)	Cholesterol: Phospholipid	Drug in Solvent
1	Low	Low	High	Low
2	Low	High	Low	Low
3	High	Low	High	Low
4	High	Low	Low	Low
5	Low	High	Low	High
6	High	High	Low	Low
7	High	Low	High	High
8	Low	High	High	Low
9	High	High	High	High
10	High	High	Low	High
11	Low	High	High	High
12	High	High	High	Low
13	Low	Low	Low	Low
14	Low	Low	High	High
15	Low	Low	Low	High
16	High	Low	Low	High

### Liposome preparation process of Aphanamixis polystachya leaf extract

Solvent injection method was used in two different manners (drug dissolved in ethanol and drug dissolved in water) to formulate the liposomal preparation. Briefly, drug extract was mixed with the phospholipid extract and cholesterol, which were dissolved in ethanol. The mixture was then injected at two different rates into water that was being stirred, using a magnet stirrer according to a pre-determined speed (Table 1). Heating was done to evaporate the remaining excess of ethanol that was in the final product. The mixture was left for a while to cool down. Similarly, the drug extract was added into the water at the beginning of the process. The phospholipid and cholesterol were dissolved in ethanol. The ethanolic solution containing phospholipid and cholesterol was then injected into the aqueous drug solution at two different injection rates, and it was being stirred at a pre-determined rate (Table 1). 10 mL of the ethanolic solution was injected into 100 mL of distilled water during preparation of the liposomal batches of *A. polystachya*.

### GC-MS Method

A Perkin Elmer model Clarus 600 T combined with single quadrupole mass spectrometer was used for GC-MS analysis. The chromatographic column was an Elite 5MS column (30 m × 0.25 mm × 0.25 µm film thickness), with high-purity helium as the gas carrier, at a flow rate of 1 mL/min. The injector temperature was 280 °C and it was equipped with a splitless injector at 20:1. The temperature was set initially to 40 °C (held for 2 min), then increased to 150 °C at 5 °C min^−1^ (held for 2 min), and further increased to 300 °C at 5 °C min^−1^ (held for 2 min). The MS ion source temperature was 200 °C and inlet line temperature at 220 °C. The scan range was set at 40 to 600 mass ranges at 70 ev electron energy and the solvent delay of 4 min. Finally, unknown compounds were identified by comparing the spectra with that of the NIST 2005 (National Institute of Standard and Technology library) and Wiley 2006 library. The total time required for analyzing a single sample was 58 min.

### Transmission Electron Microscopy (TEM)

The morphology of the vesicles from the optimized liposomal batches was investigated using an FEI Tecnai G2 TEM (FEI, Holland). Each vesicle was freshly prepared, diluted with distilled water and one drop was placed on the carbon-coated copper grid which was left to dry. A drop of osmium was used to stain lipid components. Once dry, it was loaded into the TEM and viewed at 5000 – 20,000 magnifications.

### Particle size and Zeta Potential

Particle size distribution, polydispersity index (PDI) of liposomal batches were measured using disposable polystyrene cuvettes, and zeta potential were measured using plain folded capillary zeta cells by a Malvern Zetasizer Nano ZS90 (Malvern Instruments, UK) at 25 °C. The analysis was performed in triplicate, and the average value was used from the collected data.

### Fourier Transform Infrared Spectroscopy (FT-IR)

The IR spectrum for a liposomal formulation compared to the blank was revealed using Perkin Elmer Fourier transform infrared (FT-IR) spectrum BX. KBr pellets were freshly prepared to avoid any moisture effect and the spectra of 4400 to 400 ${\text{cm}}^{-1}$ wavenumber were collected using 24 scans and 2 cm^−1^ resolution^37^.

### Behavioral study using mice model

#### Animal

Swiss albino mice (male and female) of 10 weeks’ age, weighing in the range of 40–45 g were used in this study. The animals were caged in groups and were given sufficient food and water. Temperature, humidity, and 12 hour of light cycle were maintained. This study was carried out in accordance with the principles of National Institute of Health Guide for the Care and Use of Laboratory Animals (NIH Publications No. 80–23; 1996). The experimental procedure was reviewed and approved by the institutional animal ethics research committee (project identification code- AEC-08–2018, approved on 08/07/2018) at North South University, Dhaka, Bangladesh.

#### Preparation of test materials

*Aphanamixis polystachya* leaf extract at dose equivalent to 400 mg/kg body weight of mice was given orally to evaluate its effect on mice model^26^. Later, blank liposome, leaf extract and representative liposomal formulation at a dose of 200 mg/kg were applied for disease & blank liposome group, extract group and two treatment groups of mice. The extract was dissolved using normal saline water and given orally at volume equivalent to 200 µL per mice. Similarly, olanzapine, which is used to induce dimentia in mice, was given at a dose of 15 mg/kg at volume of 200 µL.

#### Designing of the Experiment

Forty mice were divided into five groups for open field and arm maze studies [control, disease & blank liposome, treatment group 1 (Extract) and treatment group 2 (liposomal formulation of *Aphanamixis polystachya* leaf extract)] and each group contained eight mice. Water maze study was performed on four groups where disease & blank liposomes group was not used, since we did not find any significant effect of blank liposome on disease improvement:

Control group received blank liposome only orally for 14 days. Disease group received disease inducer (Dose: 15 mg/kg body weight). Olanzapine dissolved in normal saline and given daily for 14 days to induce Alzheimer’s disease in the mice.

Disease & blank liposome group received olanzapine in normal saline (200 µL/per mice) with blank liposome (200 mg/kg body weight) and given orally simultaneously for 14 days to treat Alzheimer’s disease.

The treatment (group 1) received disease inducer, i.e. Olanzapine (200 µL/per mice) and ethanolic extract of *Aphanamixis polystachya* (200 mg/kg body weight) dissolved in normal saline and given orally daily simultaneously for 14 days to treat Alzheimer’s disease.

The treatment (group 2) received disease inducer, i.e. Olanzapine (200 µL/per mice) and liposomal formulation of *Aphanamixis polystachya* extract (200 mg/kg body weight) mixed with normal saline and given orally daily simultaneously for 14 days to treat Alzheimer’s disease. The mice had undergone behavioral studies both before and after gavage of the chemicals. After the behavioral studies completed, the mice were sacrificed.

#### Open Field

To evaluate CNS disorders in rodent models, the open field test is used to determine general activity levels, gross locomotor activity, and exploration habits^38^. Rodent movements are continuously analyzed by an automated tracking system for the following parameters: distance moved, velocity, and time spent in pre-defined zones.

Mice were placed into the centre or one of the four corners of the open field and allowed to explore the apparatus for 20 min. After the 20 min test, mice were returned to their home cages, and the open field was cleaned with 70% ethyl alcohol and permitted to dry between tests. To assess the process of habituation to the novelty of the arena, mice were exposed to the apparatus for 10 min.

#### Arm Maze

Radial arm maze method is used as a tool to examine the effect of pharmacological compounds on memory^39^. Here, mice were trained, so that it can learn and explore the maze properly. On the last day of training, the baits were reduced by half from eights arms to a single arm in three consecutive days of training. Using its reference memory and spatial learning capacity, mice has to find out the target arm within 15 min from the time it has been placed in to the center of the maze. After 14 days of treatment we kept mice deprived of food for a night and then on next day we continued the experiment keeping food in a particular target arm to observe the reference and spatial learning capacity of mice.

#### Water Maze

The widely used behavioral neuroscience procedure for rodents is Morris water maze test. It is mainly used behavioral to study spatial learning and memory. Therefore, any damage to cortical regions is assessed through the accurate study of learning, memory, and spatial working^40–43^.

In this procedure, the rat is placed in a large circular pool and it is supposed to find an invisible or visible platform that allows it to escape the water through various cues.

#### Data Analysis

GraphPad prism 6 was used to analyze the data. Statistical analysis was performed by one way ANOVA. Where **** means p ≤ 0.0001 *** means p ≤ 0.001 and ** means p ≤ 0.01 and number of animal in each group n = 8.

### *In-vivo* anti-inflammatory study

#### Animals

Twenty-four Long-Evans female rats average weight of 185–195 gm were obtained from North South University’s animal house. All animals were housed at ambient temperature and relative humidity. The animals were fed with standard diet and water and were deprived of food overnight before the experiment. The experimental procedure was reviewed and approved by the institutional animal ethics research committee (project identification code- AEC-09-2018, approved on 04/08/2018) at North South University, Dhaka, Bangladesh.

#### Methods

Twenty-four rats were weighted and randomized into four groups; each group contained six rats. Group 1 was used as a positive control where 0.1 mL of 1% solution of carrageenan in 0.9% saline was used, Group 2 was used as an extract group where *Aphanamixis polystachya* leaf extract (80 mg/kg body weight of rat) was used, Group 3 was used as formulation group where liposomal formulation of *Aphanamixis polystachya* extract (80 mg/kg body weight of rat) was used, Group 4 was used as a standard group where diclofenac sodium was used (10 mg/kg body weight of rat) as a standard.

All rats were kept for one week to acclimatize to the laboratory conditions. Initial paw size of all rats was taken by plethysmometer for each group and recorded. The first dose of the control, extract, formulation and standard group were administered intraperitoneally (IP) for all rats. After one hour, the second dose was administered intraperitoneally (IP) for all four groups. 0.1 mL of 1% solution of carrageenan in 0.9% saline was injected subcutaneously into the sub-plantar region of the left hind paw of all rats one hour after the second dose.

The first day, after 3 or 5 hours of carrageenan administration, the paw size of all rats was taken by plethysmometer. From day 2 to day 4, control, extract, formulation, and standard drug were administered in the same way as mentioned above. After 3 hours of the second administered dose, the paw size of all rats was taken by plethysmometer.

### Data analysis

The data were expressed as mean ± standard deviation (SD). The percentage inhibition of edema was calculated for each group in comparison with the control group.

The percentage (%) inhibition of edema was calculated using the formula -

$$ \% \,{\rm{inhibition}}=\frac{{\rm{To}}-{{\rm{T}}}_{t}}{{\rm{To}}}\times 100$$

Where T_0_ is paw volume of positive control group and T_*t*_ is the paw volume of treatment group

## Results

### GC-MS analysis of *Aphanamixis polystachya* of leaf extract

**Figure 1:**
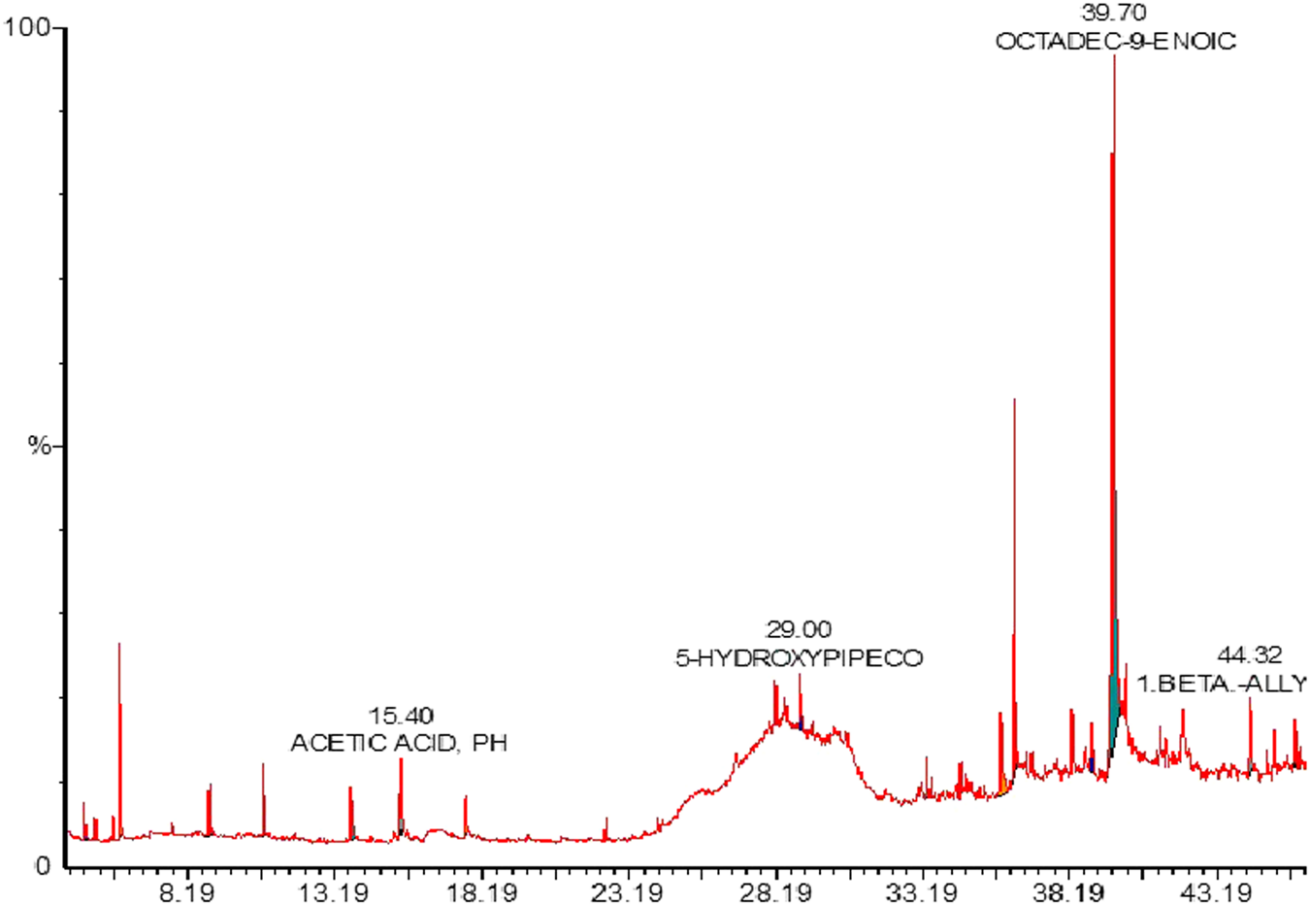
GC-MS chromatogram of the leaf extract of *Aphanamixis polystachya*.

The major ingredients present in *Aphanamixis polystachya* leaf extracts are listed in Table 2, (GC-MS chromatogram shown by Fig. 1) including 2-Pentanone, higher fatty acids (Octadec-9-enoic acid, 5-hydroxypipecolic acid, hexadecanoic acid), 2-hydrazino-2-imidazoline and beta-elemene etc. 2-Pentanone and Beta-elemene found to have poor solubility in water.

**Table 2:** List of major components present in the leaf extract of *Aphanamixis polystachya*.

No.	Name	RT	Area %	N. Area %
1	2-PENTANONE	5.88	14.620	51.080
2	1,2-CYCLOHEXANEDIOL	13.76	4.120	14.390
3	ACETIC ACID	15.40	4.050	14.140
4	2-HYDRAZINO-2-IMIDAZOLINE	17.60	3.410	11.91
5	BETA-ELEMENE	22.40	0.400	1.390
6	5-HYDROXYPIPECOLIC ACID	29.00	1.880	6.560
7	9-HEXADECENOIC ACID	35.88	5.660	19.770
8	HEXADECANOIC ACID	36.32	8.830	30.840
9	OCTADEC-9-ENOIC ACID	39.72	28.630	100.000
10	1.BETA.-ALLYLPERHYDRO-2.ALPHA,5,5,8 A,BETA TETRAMETHYL-TRANSNAPHTHALEN-2BETA-OL	44.32	1.320	4.600
11	CYCLOPENTADECANONE	44.86	4.020	14.020
12	TRIDEC-4-EN-2-YNAL	50.06	3.710	12.960

Beta-elemene is used as a traditional medicine in China. It is considered as a novel anticancer agent and used for the treatment of melanoma, glioblastoma, breast cancer, lung cancer, esophageal cancer and pancreatic cancer^44–46^. Importantly, it is reported to down-regulate the inflammatory cytokines in optic neuritis in an animal model of multiple sclerosis^47^. Besides, beta elemene exhibit strong antioxidant and anti-inflammatory property and hence improved the outcome of atherosclerosis in an animal model^48^. A recent elegant study reported that beta elemene improved traumatic brain injury in rats by reducing inflammation^49^.

2-pentanone is a flavoring volatile ketone compound usually found in fruits and vegetables including banana, cultivated strawberries and carrot. It inhibits prostaglandin production and demonstrates COX-2 inhibitory effect in colon cancer cells^50^. Inflammation is critically involved in neurodegenerative disorders including Alzheimer’s disease and known to aggravate the disease outcome^51^. Ketone bodies are known for long to improve cognitive functions in neurodegenerative disorders including stroke^52–54^.

5-hydoxypipecolic acid is responsible for neurotransmission in brain through lysine pathways^45,55,56^. Lysine is catabolized by pipecolic acid oxidase with the help of peroximal enzyme present in mitochondria. Hyperlysinemia is a rare disease caused by the deficiency of enzyme 2-amino adipic semialdehyde synthase in lysine degradation. Pipecolic acid interferes with the degradation of lysine through other pathway and decrease the concentration of lysine in mitochondria^57^.

### Particulate Characterization

#### Transmission Electron Microscopy (TEM)

**Figure 2:**
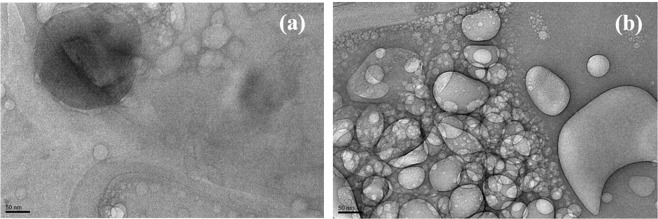
TEM micrographs of *Aphanamixis Polystachya* loaded liposomes.

Transmission Electron Microscopic data (Fig. 2) suggests that *Aphanamixis Polystachya* loaded liposomes were in the nanosize range, which was also evident by the particle size distribution data obtained using Malvern Zetasizer (Table 3 & Figure S1 in supplementary data).

**Table 3:** Particle size distribution data and polydispersity index for the liposomal batches of *A. polystachya*.

Sample	Injection Rate (mL/sec)	Stirring Speed (rpm)	Cholesterol: Phospholipid	Drug in Solvent	Mean Particle Size	Particle Size ± SD	Mean PDI	PDI ± SD
1	Low	Low	High	Low	166.9	14.3	0.47	0.04
2	Low	High	Low	Low	174.4	0.2	0.14	0.03
3	High	Low	High	Low	147.5	3.7	0.26	0.07
4	High	Low	Low	Low	144.4	3.1	0.24	0.02
5	Low	High	Low	High	177.3	65.0	0.43	0.07
6	High	High	Low	Low	157.2	1.4	0.14	0.02
7	High	Low	High	High	171.4	7.0	0.34	0.01
8	Low	High	High	Low	149.8	1.8	0.23	0.03
9	High	High	High	High	145.6	2.3	0.29	0.04
10	High	High	Low	High	141.6	4.7	0.35	0.03
11	Low	High	High	High	163.1	0.4	0.37	0.02
12	High	High	High	Low	176.4	3.0	0.28	0.04
13	Low	Low	Low	Low	117.6	5.3	0.29	0.02
14	Low	Low	High	High	132.7	4.2	0.35	0.03
15	Low	Low	Low	High	144.8	18.4	0.33	0.02
16	High	Low	Low	High	146.7	3.7	0.28	0.04

#### Fourier Transform Infrared Spectroscopy (FT-IR)

**Figure 3:**
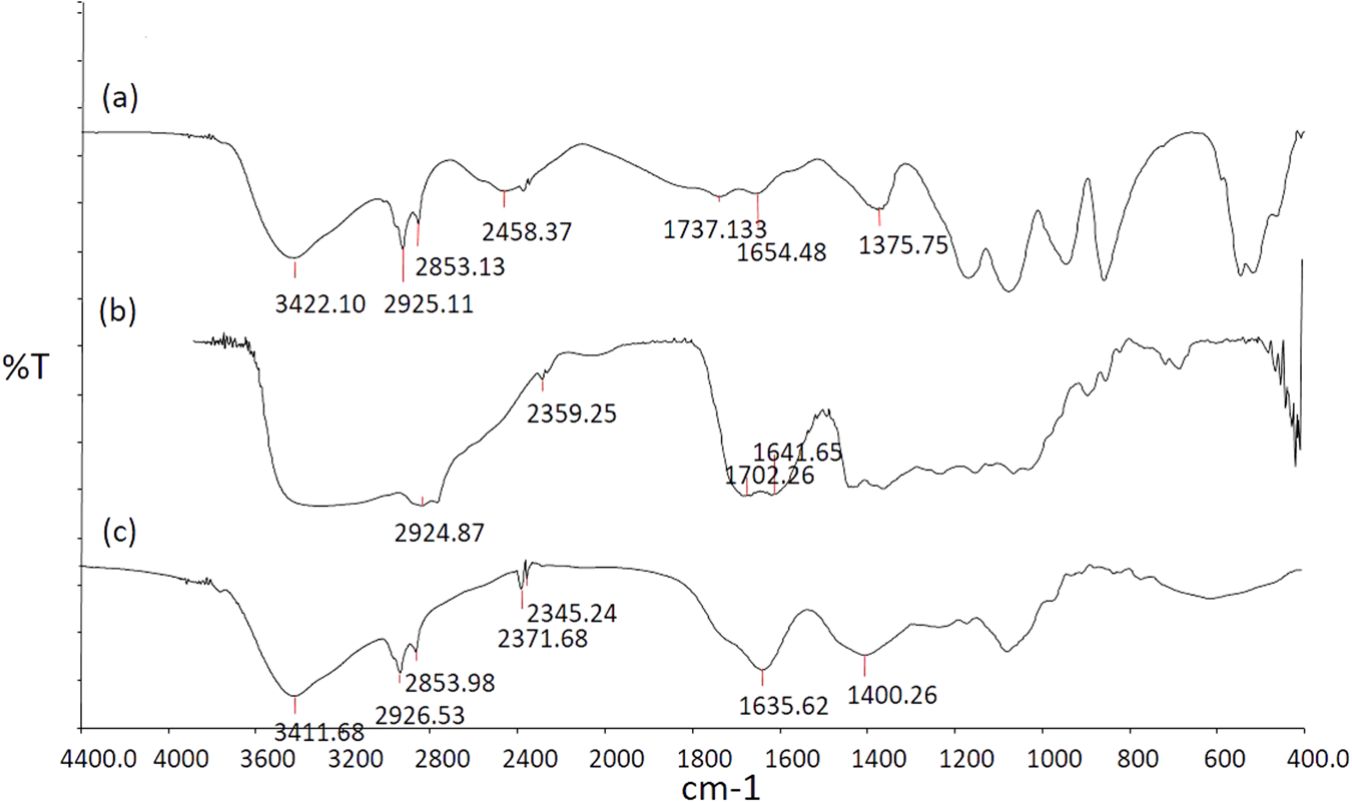
FTIR analysis of (**a**) Blank liposomal formulation, (**b**) pure *Aphanamixis polystachya* leaf extracts and (**c**) representative liposomal batches of *Aphanamixis polystachya*.

The energy of the IR region induces stretching or bending of the bonds in different molecules. Thus, such vibrational frequencies are recorded in the IR spectroscopy. The FT-IR spectra for liposomal batch and blank liposomal formulation are found in Fig. 3. From the spectra of the blank liposomal formulation, we found vibrational band with a characteristic peak for OH group at 3422 cm^−1^. Peaks at 2925 and 2853 cm^−1^ were attributed to asymmetric and symmetric stretching of CH_2_ group of phospholipids. Carbonyl functional groups showed characteristic peaks at 1737 and 1654 cm^−1^. It was suggested that the terminal methyl group showed a vibrational frequency at 1376 cm^−1^^58^. Hydroxyl and carbonyl functional groups showed clear vibrational bands in different extracted chemical compounds of *A. polystachya* at wavenumbers of ~3430 and 1730 cm^−1^, respectively^59,60^. This liposomal formulation containing *A. polystachya* leaf extract showed small changes in the CH_2_ band at 2926 and 2854 cm^−1^. It is speculated that, C=O functional groups for the phospholipids and the extract showed one distinct peak at 1635 cm^−1^. Hydrogen bonding is possible for hydroxyl functional groups and that was attributed to the change in of the band to be 3412 cm^−1^.

#### Physical stability

**Figure 4:**
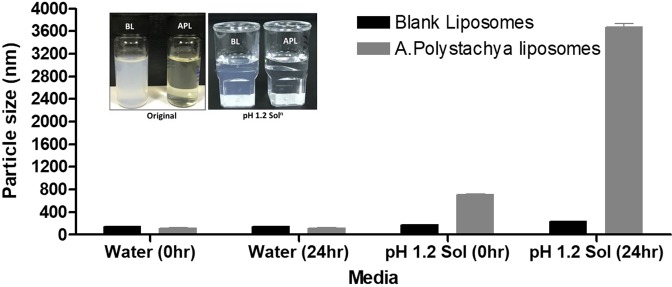
Appearances and particle size of blank liposomes and *Aphanamixis Polystachya* loaded representative liposomal formulation at 0, 24 hr time in water and gastric media. Data are represented as mean ± SD, n=3.

Blank liposomes and *Aphanamixis Polystachya* loaded representative liposomes were diluted with gastric media (pH 1.2) to investigate the survival of liposomes in gut. The appearance of the dispersion (Fig. 4) was assessed visually, which suggested liposomal precipitation of *Aphanamixis Polystachya* loaded representative batch^61^. This was further evidenced by the lager particle size of the batch as it was increased from 117.6 to 716.4 nm in gastric media at 0 time to 3672.67 nm within 24 hrs time (Fig. 4). However, the dispersion data at 4 hrs (Table S1 in supplementary data) showed that there was not any shuttle change in particle sizes increase. Particle size distribution data also showed that no significant difference in particle size for blank liposome batch before and after pH stability study at 4 hours (maximum transit time in the stomach). There were no significant phase changes and/or precipitations were recorded for blank liposomes (Fig. 4). As *Aphanamixis Polystachya* loaded liposome produced the finest emulsions but a limited particle size difference between them is not likely to determine the differences between their performances *in vivo* from the oral dosage form. The overall data from the pH stability study suggest that no physical changes such as precipitation or degradation were observed for blank liposome even after 24 hrs.

#### Particle size distribution data

Particle size distribution data (Table 3) showed that average particle size of liposome batches were in the range of 117–180 nm. Polydispersity index (PDI) value found for all liposomal batches were below 0.5 (Table 3).

#### Effect of process parameters and material attributes on average particle size

**Figure 5:**
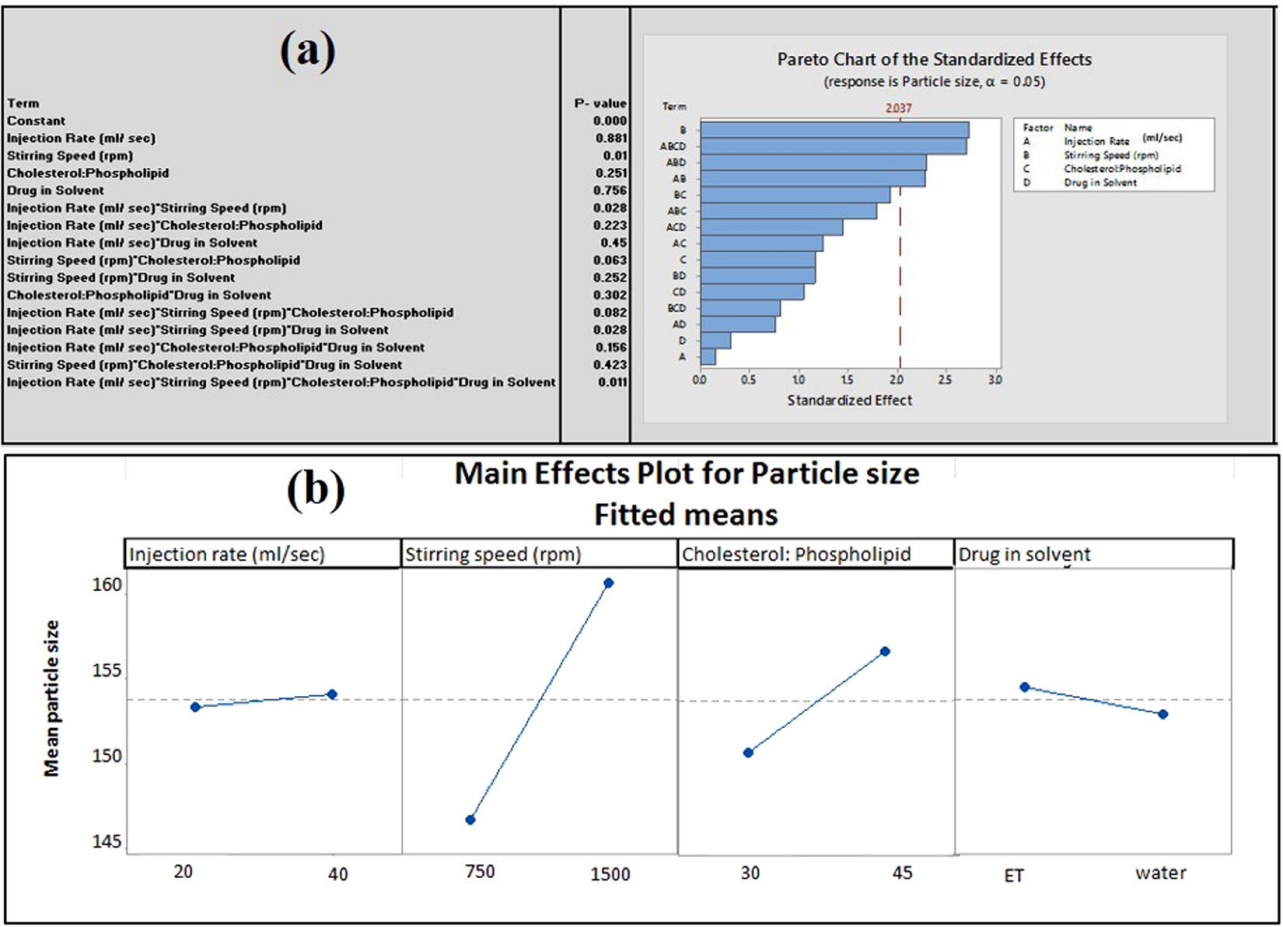
(**a**) Pareto plot showing the effect of process parameters (PP) and material attributes (MA) on average particle size of liposome. (**b**): Main Effect plot of PP and MA on the average particle size of liposomes [Where injection rate: 20 = 10 mL was injected at 20 sec. and 40 = 10 mL injected at 40 sec., Cholesterol: Phospholipid value 30 indicates cholesterol: phospholipid = 1: 3, Cholesterol: Phospholipid value 45 indicates cholesterol: phospholipid = 1: 4.5].

The pareto plot for the mean particle size with p-values for the different factors and interaction in rank order (Fig. 5a) show that the process parameters and material attributes demonstrate significant (p ≤ 0.05) effects and interactions on the average particle size of liposomes (Fig. 5a).

Stirring speed showed to have a most significant effect on average particle size of *A. polystachya* loaded liposome (p ≤ 0.01) (Fig. 5b). Low, stirring speed allowed the development of nanoparticles in the range of 130-150 nm. Low, stirring speed (750 rpm) used in this study might be the optimum level for the liposomal formulation of *Aphanamixis polystachya*. Liposomal batches processed at high stirring speed (1500 rpm) exhibit larger particle size compared to batches processed at low stirring speed (Fig. 5b). Other parameters did not show any significant effect individually on the average size of liposome.

#### Effect of process parameters and material attributes on Polydispersity Index

**Figure 6:**
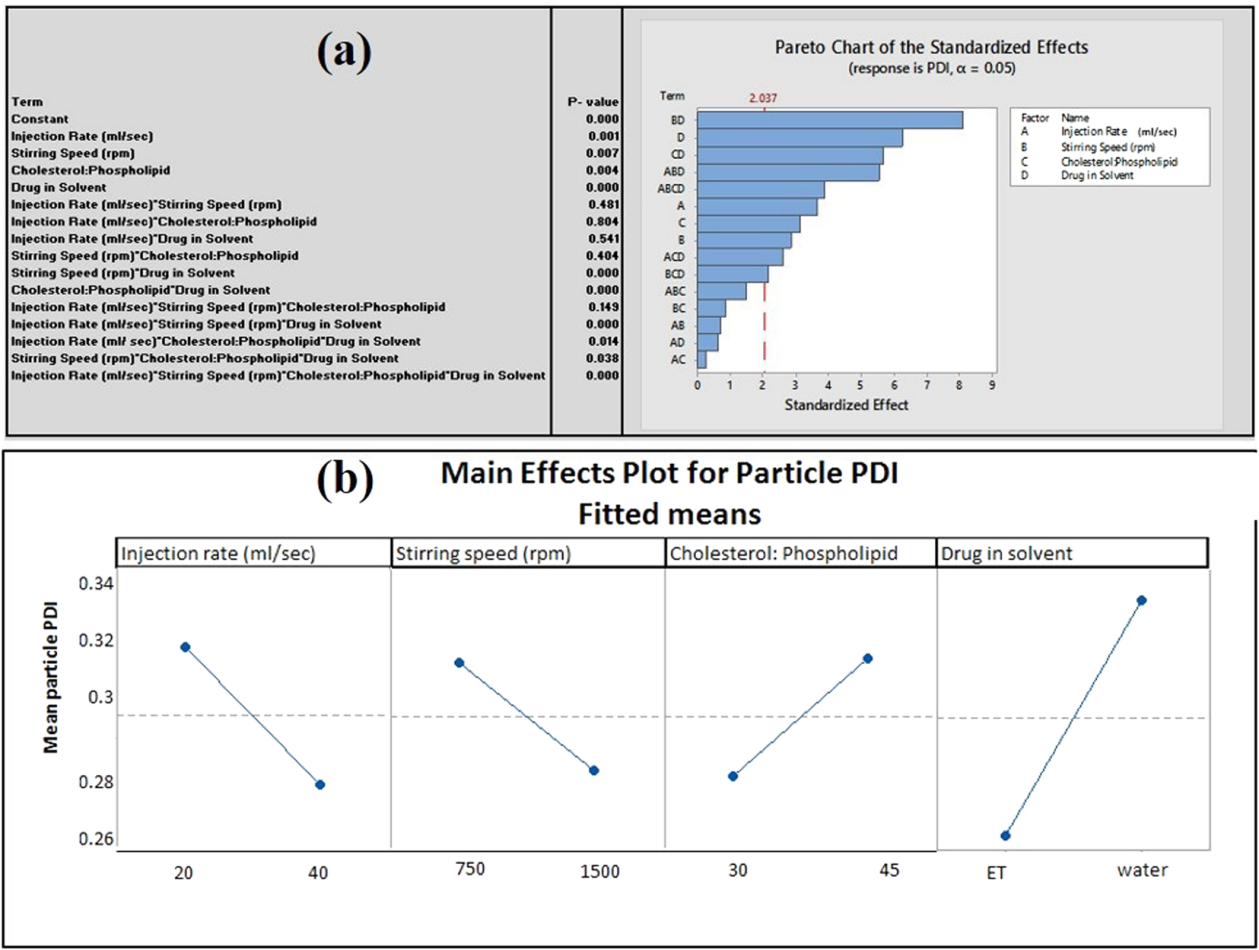
(**a**) Pareto Chart of PP and MA on PDI of liposome, (**b**): Main Effect plot of PP and MA on PDI of liposome [Where injection rate: 20 = 10 mL was injected at 20 sec. and 40 = 10 mL injected at 40 sec., Cholesterol: Phospholipid value 30 indicates cholesterol: phospholipid = 1: 3, Cholesterol: Phospholipid value 45 indicates cholesterol: phospholipid = 1: 4.5].

Individual process parameters and material attributes showed significant effect on PDI value (p ≤ 0.01), where drug in solvent is having a most significant effect on PDI value of liposomal batches (p ≤ 0.001) (Fig. 6a). Liposomal batches of *A. polystachya* leaf extract dissolved in ethanol exhibited narrow size distribution compared to batches prepared in water (Fig. 6b). This phenomenon probably related to the different degree of supersaturation achieved when different solvent was used during liposome preparation of *A. polystachya* leaf extract*. A. polystachya* leaf extract probably less soluble in water compared to ethanol, also phospholipid is less soluble in water. Therefore, liposome batches prepared using water having high degree of supersaturation leading to spontaneous crystallization at labile zone^62^. This may be result in polydisperse particles for the liposome batches of *A. polystachya* prepared using water.

#### Interactions of process parameters and material attributes and its impact on average particle size

**Figure 7:**
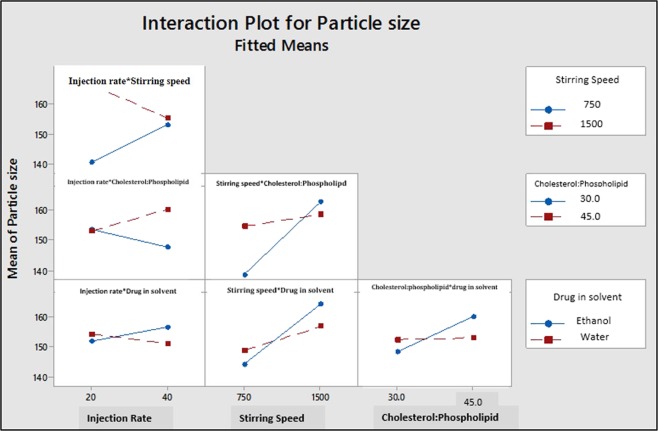
Two-way Interaction plot of PP and MA for particle size [Where injection rate: 20 = 10 mL was injected at 20 sec. and 40 = 10 mL injected at 40 sec. Cholesterol: Phospholipid value 30 indicates cholesterol: phospholipid = 1: 3, Cholesterol: Phospholipid value 45 indicates cholesterol: phospholipid = 1: 4.5].

The most significant two-way interaction was observed between injection rate and stirring speed (p ≤ 0.028) (Fig. 5b) which suggests that liposome batches processed at low stirring speed and faster injection rate generates small average size of liposomes (Fig. 7).

#### Interactions of process parameters and material attributes and its impact on polydispersity index (PDI)

**Figure 8:**
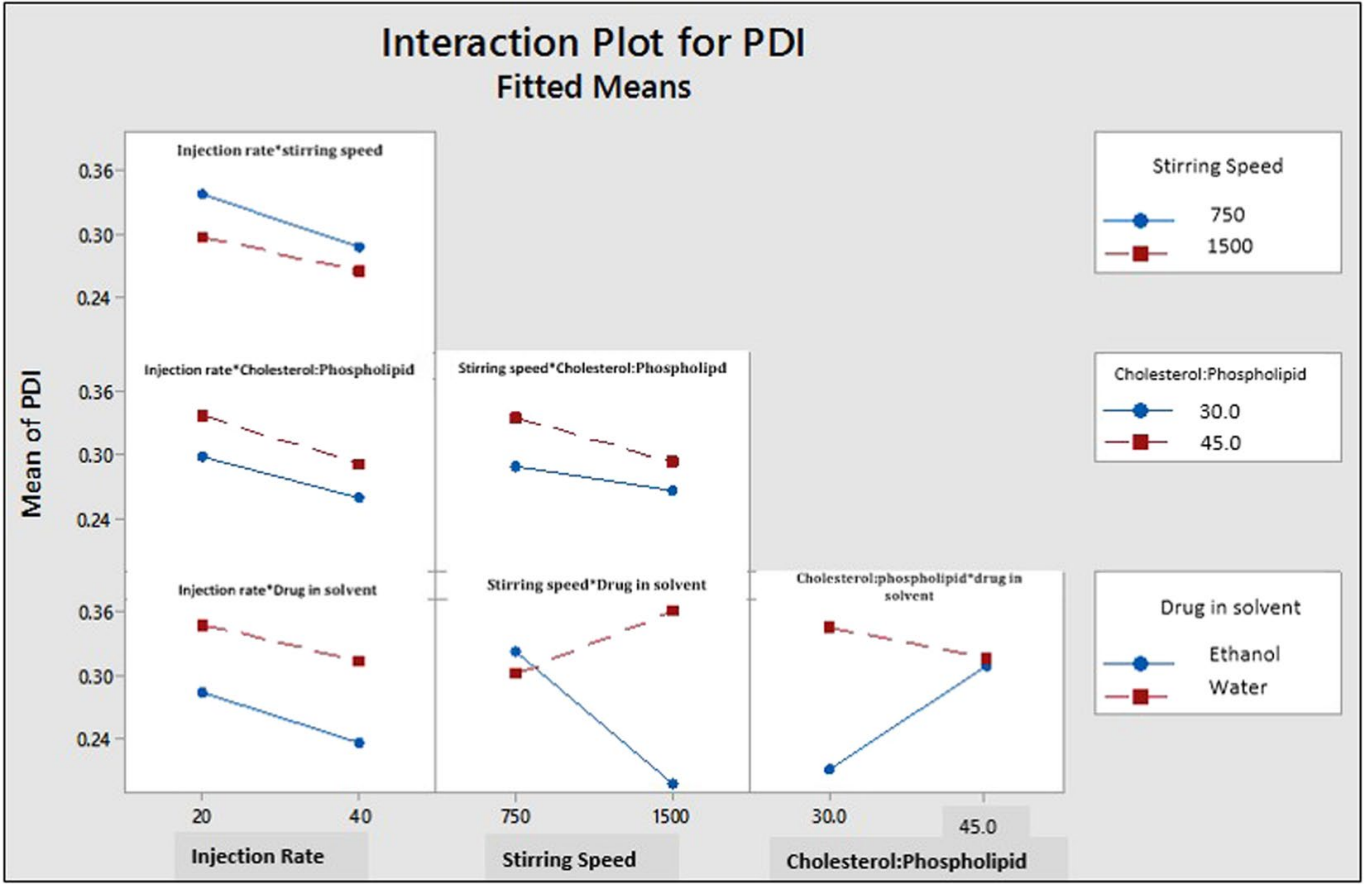
Two-way Interaction Plot of PP an MA for PDI [Injection rate: 20 = 10 mL was injected at 20 sec. and 40 = 10 mL injected at 40 sec., Cholesterol: Phospholipid value 30 indicates cholesterol: phospholipid =1:3, Cholesterol: Phospholipid value 45 indicates cholesterol: phospholipid = 1: 4.5].

Major two-way interactions were observed between stirring speed with the drug in the solvent, and cholesterol to phospholipid ratio with the drug in the solvent. (p ≤ 0.000) (Figs. 6a and 8).

Results suggest that low PDI was achieved when the drug was dissolved in ethanol and processed with high stirring speed and at low cholesterol: phospholipid ratio (1:3). These results suggest that interaction between PP and MA is critical and have significant impact on the quality attributes (particle size, and PDI) of liposomes.

### *In-vivo* behavioral study of *A. polystachya* liposomes using different mice model

**Figure 9:**
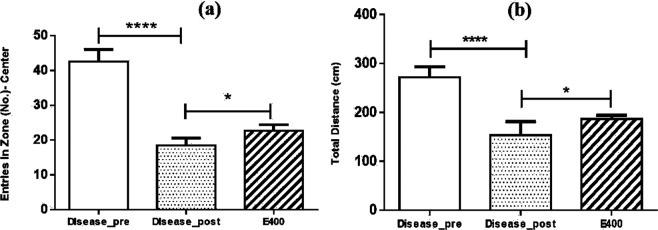
Open field studies of *Aphanamixis polystachya* leaf extract showing the improvement in the locomotor activity (Where **** means p ≤ 0.0001 and * means p ≤ 0.05).

Open field study was performed initially of *Aphanamixis polystachya* leaf extract using 400 mg/kg body weight of mouse (Fig. 9). Results indicate a significant improvement in locomotor activity and ambulatory performance for mice treated with *Aphanamixis polystachya* leaf extracts compared to post disease group.

**Figure 10:**
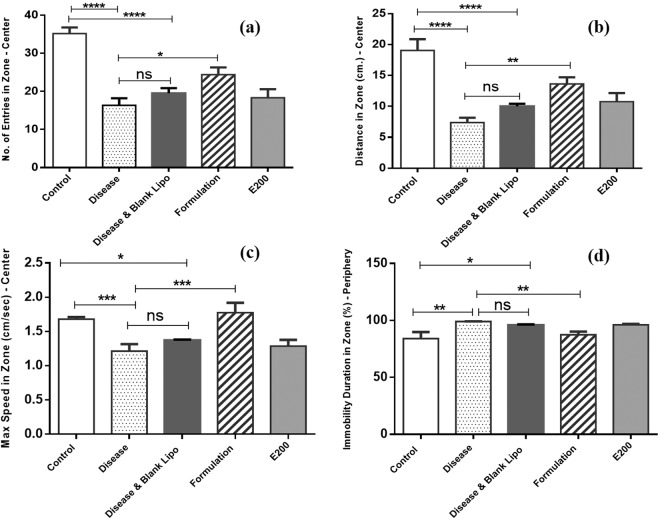
(**a**) Number of entries into the central zone of open-field. (Where **** means p ≤ 0.0001 and ** means p ≤ 0.01), (**b**): Distance in central zone travelled by different group of mouse in open-field. (Where **** means p ≤ 0.0001 and ** means p ≤ 0.01), (**c**): Maximum speed into central zone for mouse groups in open-field study. (Where ** means p ≤ 0.01), (**d**): Immobility duration of mice in periphery region of open-field study (Where ** means p ≤ 0.01).

**Figure 11:**
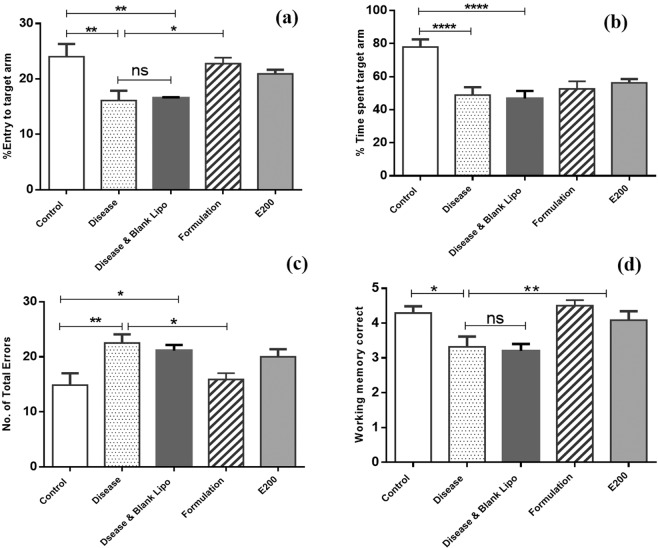
(**a**) Percent Time spent into target arm, (**b**): Percent of entries into the target arm. (Where **** means p ≤ 0.0001, ** means p ≤ 0.01 and * mean p ≤ 0.05), (**c**): Working memory correction in arm maze study, (**d**): Number of total errors in arm maze study. (Where ** means p ≤ 0.01 and * means p ≤ 0.05).

**Figure 12:**
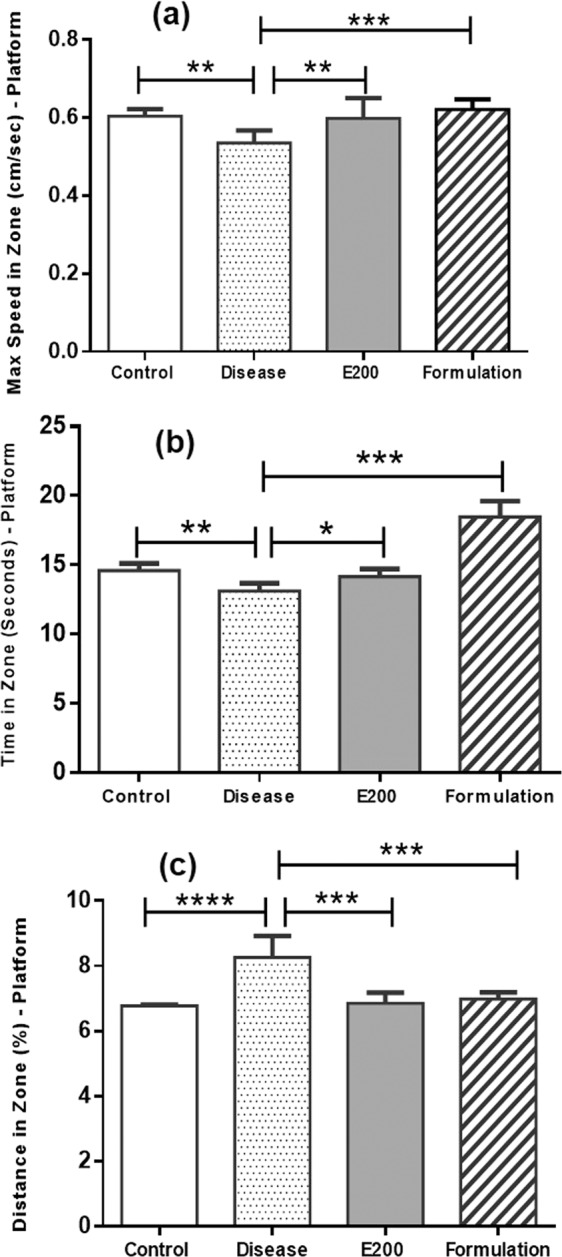
(**a**) Maximum speed to reach the platform during water maze study; (**b**) Time spent on platform for different mice groups in water maze study; (**c**) Distance travelled to platform by different mouse groups during water maze study. (Where **** means p ≤ 0.0001, *** means p ≤ 0.001, ** means p ≤ 0.01 and * mean p ≤ 0.05).

*Aphanamixis polystachya* leaf extract showed significant improvement of the locomotor activity of mice, therefore we aimed to develop liposomal formulation of this extract to investigate its performance or ability to improve the effect of this extract against dementia induced mice. However, extract concentration at 400 mg/ kg body weight of mouse was difficult to prepare as liposomal preparation <300 nm size range. This is probably high extract concentration leading to spontaneous crystallization with wide size range of liposomes. Therefore, we used 200 mg concentration of liposomal preparation. This formulation was investigated on dementia induced mice using open field, arm maze, and water maze methods (see Fig. 10, Fig. 11 & Fig. 12).

#### Open Field

Entries in central zone are significantly higher (p ≤ *0.01*) for mice treated with liposomal formulation of *Aphanamixis polystachya* compared to disease, disease & blank liposome and higher than the E200 (Extract) group (Fig. 10a). This is probably anxious mice will have lower locomotion activity towards central zone, and therefore liposomal formulation improves locomotion activity in dementia induced mice than disease and E200 groups.

Mice group treated with liposomal formulation (Fig. 10b) showed significant improvement (p ≤ *0.01*) in their ambulatory behavior compared to disease and higher than extract group, which is probably due to decreased anxiety level compared to other groups.

Maximum speed into central zone was significantly higher (p ≤ *0.01)* for formulation group compared to both disease and E200 groups (Fig. 10c). This is possibly related to the improvement of locomotion and ambulatory behavior of this group of mice compared to other groups.

Immobility duration (%) in peripheral zone is decreased significantly (p ≤ *0.01*) for formulation group compared to disease group that indicates static state or immobility has been decreased in formulation group mice comparatively to other groups (Fig. 10d). Locomotive action becomes improved among mice treated with liposomal formulation of *Aphanamixis polystachya* leaf extract. However no significant improvement was observed for the mice group treated with blank liposome (Disease & Blank Lipo group) compared to disease group (Fig. 10).

#### Arm Maze Result

To determine total time mice spent in the target arm was another important parameter to know about the improvement in memory of dementia induced mice model after treating with *Aphanamixis polystachya* extract (E200) and liposomal formulation. The rate of entries to target arm, result (Fig. 11a) indicates that it has been increased significantly (p ≤ 0.05) for formulation given group compare to disease group. Formulation given group and E200 group spent more time in target arm compared to disease group; however, difference was not significant (Fig. 11b).

Figure 11c, indicates that working memory correction of the formulation group was significantly improved (p ≤ *0.01*) compared to disease group. It suggests that the learning capacity or memory functions of mice treated with liposomal formulation of *Aphanamixis polystachya* leaf extract was improved compared to other groups and may have positive impact on cognitive functioning of mice. Figure 11d, shows that total errors also decreased significantly (p ≤ *0.05)* for dementia induced mice treated with liposomal formulation compared to disease group. In this study also mice group (Disease & Blank Lipo group) treated with blank liposome did not show any significant effect compared to disease group (Fig. 11).

#### Water Maze

Morris water maze test was used to find out the significant differences between post disease group, E200 group (extract group) and liposomal formulation group. In previous studies (Open Field and Arm Maze) mice group treated with blank liposome showed no significant improvement therefore we did not run disease & blank liposome group in water maze study.

We investigate the gradual recovery from disease state to possibly recovered state using extract and liposomal formulation of *Aphanamixis polystachya* leaf extract. The objective of this study was to make the mice able to find out the platform that demonstrates the memory and learning of different mice groups. If the mice have any hippocampus injury or abnormalities due to the disease that we have induced in them, they might show irregular movement to find out the previous position or would have roam around in the other quadrant of the pool apart from the real one. We have observed comparatively better motor and visual activity among mice model treated with liposomal formulation compared to post-disease and extract groups. In this study Morris water maze test was performed on following parameters:

##### Maximum speed in zone (platform).

Maximum speed to find out hidden platform in spatial experiment showed significantly better performance for liposomal formulation group (p ≤ 0.001) compared to both extract (E200) group and post disease group (Fig. 12a). Result suggest that as the day passed on and our trial time increased we found gradual improvement in finding the previous pool position with maximum speed and decreased latency or escape time.

##### Time in zone (seconds) platform.

Both extract (p ≤ 0.05) and liposomal formulation of *Aphanamixis polystachya* given group (p ≤ 0.001) showed significant retention of memory compare to disease group. Moreover, formulation group exhibit significantly better performance (p ≤ 0.01) compared to mice treated with extract group (Fig. 12b).

##### Distance in zone percent (platform).

Spatial learning of platform position and swimming patterns in different trials have been processed by the measurement of cumulative distance to the platform. Mice group treated with liposomal formulation of *Aphanamixis polystachya* leaf extract took less distance in travel to reach the target quadrant contains the platform. After inducing disease, mice found difficulties in recognizing or finding out the target zone of platform, however as the treatment goes on we saw that distance in path length and escape latency decreases significantly for treated mice [extract group (p ≤ 0.001) & formulation group (p ≤ 0.001)] compared to disease group (Fig. 12c). However, in this case both extract and formulation group exhibit similar improvement after treatment.

### *In-vivo* anti-inflammatory study

**Figure 13:**
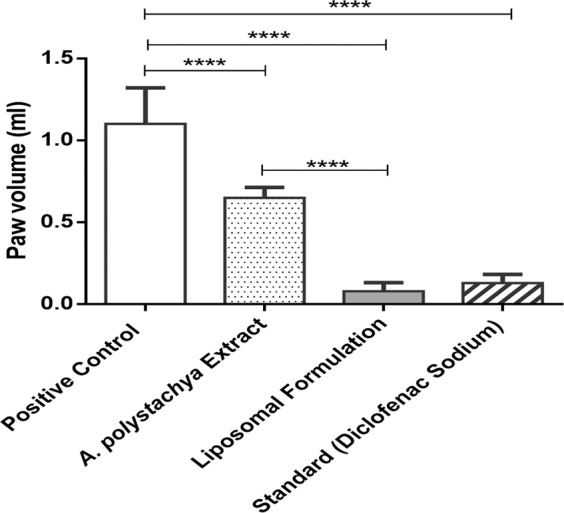
Anti-inflammatory study of *A. polystachya* leaf extract and its liposomal formulation after 4 days (Mean value with 95% CI, where **** means p ≤ 0.0001).

In this study *Aphanamixis polystachya* leaf extract and liposomal formulation showed significant anti-inflammatory activity (p ≤ 0.001) against acute edema compared to that of the positive control group (Fig. 13, and Table S2 in supplementary data). It was observed that the *A. polystachya* liposomal formulation exhibit significantly higher reduction in paw edema (p ≤ 0.001) compared to extract group and higher than standard used during anti-inflammatory study. This study suggests that *A. polystachya leaf* extract has strong anti-inflammatory activity, and this activity nearly doubled when given through liposomal delivery.

### Discussion

GC-MS data indicates presence of some novel compounds (2-Pentanone, beta-elemene, 5-hydroxypipecolic acid etc.), which were also observed by previous researcher and found to have different pharmacological properties. It is hypothesized that these compounds may possess some chemical groups which are active against CNS inflammation.

Individually PP and MA showed effect at different levels compared to the interaction between PP and MA. For example, in isolation Injection rate did not show any significant effect on average particle size of liposomes, however injection rate and stirring speed exhibit significant interaction on average particle size of liposome. Results suggest that interactions between PP and MA found to have more impact on average particle size and polydispersity of liposome batches compared to PP and MA in isolation.

Previous research suggests that neurodegenerative disease (dementia, Alzheimer’s, Parkinson’s etc.) can be treated through few possible pathways including – AChE inhibition, enhancement of cholinergic activity in CNS, anti-inflammatory and antioxidant activity. Behavioral study data suggests that improvement in the memory function, locomotor activity and ambulatory performance of dementia induced mice probably related to the strong anti-inflammatory effect evaluated for *A. polystachya* leaf extract. This effect along with its antioxidant activity observed by previous researcher may have reduced the CNS inflammation of dementia induced mice model. It is hypothesized that compound found in the *A. polystachya* leaf extract may have some phytoconstituents or functional groups which work on the 5-LO pathway, thus inhibiting the ability of the inflammation response. However, we have to work further to confirm the mechanism of actions since CNS inflammatory response is one of the major etiological factor in triggering Alzheimer’s disease.

If *Aphanamixis Polystachya* loaded liposomes are exposed to low pH media, the particle sizes are increased. The data after 24hrs showed significant increase in the particles sizes. To investigate the rate of increase, we have measured the particle size at 4 hours (maximum transit time in the stomach) and found the very slow increase in particle sizes within the time. Therefore, we think that phospholipid used to prepare liposome were involved in the structural part only and we believe that free phospholipid that might leach from liposome structure will be insignificant to elucidate a placebo effect. In addition, Priprem *et al*. published an article^63^, which showed the use of oral quercetin liposomal delivery systems in rats. Anxiolytic and cognitive-enhancing effects of quercetin liposomal systems were subjected to elevate plus maze and morris water maze tests (they have included blank liposomes in this study). Both conventional and quercetin liposomes showed anxiolytic and cognitive-enhancing effects.

Liposomal delivery of *A. polystachya* leaf extract demonstrates significant improvement of the behavioral characteristics in dementia induced mice even compared to extract group, which is possibly related to the increase permeability of extract through the BBB.

## Conclusion

Liposome drug delivery system of *A. polystachya* was successfully produced with preferable quality attributes (average particle size & PDI). DoE study showed that quality attributes were more dependent on the interactions between PP and MA compared to individual effect of PP and MA. The stirring speed was found to be the most critical process parameter affecting the average particle size of the liposome. Drug in the solvent was a critical material attribute which showed a significant effect on the PDI of the liposome. GC-MS data indicates presence of some major compound which found to have marked impact on behavioral study of dementia induced mice model. Anti-inflammatory study result suggests that improvement in the behavioral study probably related to the strong anti-inflammatory effect of *A. polystachya* extract*. In-vivo* studies for liposomal drug delivery of *A. polystachya* leaf extract showed significantly better performance compared to extract group.

## Supplementary information

Supplementary file.
