# Supplementary material for: Liposomal drug delivery of Aphanamixis polystachya leaf extracts and its neurobehavioral activity in mice model
Source: Sci Rep. 2020 Apr 24;10:6938. doi: 10.1038/s41598-020-63894-9 (PMC7181877; doi:10.1038/s41598-020-63894-9)
Supplement: Supplementary file 1 — Supplementary file. [file 41598_2020_63894_MOESM1_ESM.docx]

**Liposomal drug delivery of *Aphanamixis polystachya* leaf extracts and its neurobehavioral activity in mice model**

Mohammad H. Shariare^1^*, Mahbubur Rahman^1^, Shamshad R. Lubna^1^, Reeti S. Roy^1^, Joynal Abedin^1^, Akbar L. Marzan^1^, Mohammad A. Altamimi^2^, Syed Rizwan Ahamad^3,4^, Ajaz Ahmad^5^, Fars K. Alanazi^2^, and Mohsin Kazi^2^*

^1^Department of Pharmaceutical Sciences, North South University, Dhaka, Bangladesh

^2^Department of Pharmaceutics, College of Pharmacy, King Saud University, Riyadh, Kingdom of Saudi Arabia.

^3^Central Laboratory, College of Pharmacy, King Saud University, Riyadh, Kingdom of Saudi Arabia.

^4^Department of Pharmaceutical Chemistry, College of Pharmacy, King Saud University, Riyadh, Kingdom of Saudi Arabia

^5^Department of Clinical Pharmacy, College of Pharmacy, King Saud University, Riyadh, Kingdom of Saudi Arabia

**Running Title:** Liposomal delivery of *A.* *polystachya* to treat neurodegenerative disease

**Keywords:** Neurodegenerative disease, Liposome, *Aphanamixis polystachya,* dementia, anti-inflammatory

***Corresponding Author;**

*Mohsin Kazi, PhD

Associate professor

Department of Pharmaceutics, King Saud University

Riyadh-11451, Saudi Arabia, Tel: +966 (0) 114677372, Fax: +966 (0) 114676295

Email: [mkazi@ksu.edu.sa](mailto:mkazi@ksu.edu.sa)

And

*Mohammad Shariare, PhD

Email: mohammad.shariare@northsouth.edu

**Particle size distribution data:**

**Figure S1:** Particle size distribution data for *A. polystachya* liposome batch

**Table S1**: The particle size of blank liposomes and *Aphanamixis Polystachya* loaded representative liposomes at 0, 4 and 24 hr time in water and gastric media. Data are represented as mean±SD, n=3.

| Media used | Particle size Z. Ave (nm) | | | | | |
| --- | --- | --- | --- | --- | --- | --- |
|  | **Blank Liposomes** | | | ***A.Polystachya* loaded liposomes** | | |
|  | 0 hr | 4 hr | 24 hr | 0 hr | 4 hr | 24 hr |
| Water | 142.03±2.79 | 141.99±3.58 | 146.53±4.44 | 117.60±5.30 | 119.8±4.0 | 118.09±6.12 |
| Gastric | 176.13±4.88 | 181.33±6.25 | 238.45±3.78 | 716.4±10.32 | 886.10±14.71 | 3673.6±112.4 |

***In-vivo* anti-inflammatory study:**

**Table S2:** Anti-inflammatory study of *A. polystachya leaf* extract and its liposomal formulation

| Time | Positive Control | *A. polystachya* Extract | | *A. polystachya*  Liposomal Formulation | | Standard | |
| --- | --- | --- | --- | --- | --- | --- | --- |
|  | **Paw Volume(ml)** | **Paw Volume(ml)** | **(%) Reduction** | **Paw Volume(ml)** | **(%) Reduction** | **Paw Volume(ml)** | **(%) Reduction** |
| 3 h (1 day) | 1.20±0.47 | 0.90±0.14 | 25 | 0.53±0.39 | 55.83 | 0.57±0.07 | 52.5 |
| 5 h (1 day) | 1.97±0.15 | 1.18±0.18 | 40.1 | 0.61±0.17 | 69.04 | 0.77±0.08 | 60.91 |
| 2 days | 1.76±0.32 | 0.93±0.07 | 47.16 | 0.34±0.13 | 80.68 | 0.36±0.10 | 79.55 |
| 3 days | 1.53±0.23 | 0.89±0.08 | 41.83 | 0.16±0.06 | 89.54 | 0.19±0.04 | 87.58 |
| 4 days | 1.10±0.21 | 0.65±0.06^****^ | 40.91 | 0.08±0.05^****^ | 92.73 | 0.13±0.05^****^ | 88.18 |
